# 

**Published:** 1920-11-27

**Authors:** 


De 9'20S

### THE HOSPITAL

The Modern Newspaper of Administrative Medicine and Institutional Life.

Telegraphic Address: OSPEDALE, RAND, LONDON. Telephone Number: 2734 GERRARD.

No. 1799, Vol. LXIX. | Saturday, November 27th, 1920. | PRICE TWOPENCE.

